# Subchronic Exposure to Low-Dose Chlorfenapyr and Emamectin Benzoate Disrupts Kidney Metabolism in Rats

**DOI:** 10.3390/toxics13010065

**Published:** 2025-01-20

**Authors:** Di Zhang, Xiao-Hua Song, Dan Yang, Mu-Zi Ge, Jun Qiu, Han-Qing Jiang, Yan-Yan Sun, Xiang-dong Li, Yi-Jun Wu

**Affiliations:** 1 State Key Laboratory of Integrated Management of Pest Insects and Rodents, Institute of Zoology, Chinese Academy of Sciences, Beijing 100101, China; 18754883207@163.com (D.Z.);; 2 University of Chinese Academy of Sciences, Beijing 100049, China

**Keywords:** pesticide, nephrotoxicity, oxidative stress, metabolomics

## Abstract

Residues of the pesticides chlorfenapyr (CFP) and emamectin benzoate (EMB) often coexist in the environment and can be accumulated in the body. To understand the impact of these two chemicals on health, we investigated their effect on the kidneys. In this study, rats were treated with CFP and/or EMB at low/medium/high doses of 1/3/9 mg/kg/day and 0.2/0.6/1.8 mg/kg/day, respectively, via oral gavage for 60 days. Kidneys and serum samples were collected and serum biochemistry and kidney histopathological changes were analyzed and examined. Kidney metabolome alterations were analyzed by using gas chromatography–mass spectrometry. The results showed that combined exposure to CFP and EMB elevated BUN levels and induced pathological damage, which presented as thinner renal tubular epithelial cells, an abnormal glomerular morphology, and an increased fibrotic area. CFP and/or EMB disrupted glutathione metabolism and carbohydrate metabolism, resulting in the alteration of kidney metabolomes and inducing oxidative stress in the cells of kidney tissues. In addition, CFP decreased ATP content and inhibited pyruvate PDH activity in the kidneys. These findings suggest that long-term exposure to CFP and EMB at environmentally relevant levels induce alterations in the renal metabolome, oxidative stress, and an insufficient energy supply, which may contribute to renal histopathological damage.

## 1. Introduction

Chlorfenapyr (C15H11BrClF3N2O, CFP), a novel pyrrole insecticide, is extensively utilized due to its broad-spectrum insecticidal activity and unique mechanism of action [1]. CFP is used to control malaria vectors and termites and pests in fruits and vegetables [1,2]. The widespread application of CFP has resulted in notable environmental residues in soil, water, and food products. It was reported that CFP residues in water, soil, and fruits and vegetables were 2.03 μg/L, 1.64 mg/kg, and 1.45 mg/kg, respectively [3,4,5]. Humans poisoned with CFP initially present with drowsiness, chest tightness, and fatigue, subsequently progressing to profuse sweating and fever, which eventually leads to respiratory failure [6]. Numerous cases of CFP poisoning have been reported, with a high mortality rate of up to 75% [7]. CFP can be metabolized into tralopyril in target organisms and experimental animals [8,9,10]. Tralopyril, as a potent uncoupler of oxidative phosphorylation, can disrupt energy metabolism by inhibiting ATP synthesis [11,12]. It was found that CFP can induce hepatotoxicity and intestinal inflammation [13]. CFP and its metabolite could be accumulated in the kidneys of mice [14]. However, it remains unclear whether long-term exposure to low-dose CFP in mammals causes nephrotoxicity.

Emamectin benzoate (C56H81NO15, EMB), a macrocyclic lactone insecticide derived from avermectin, is often applied with CFP in combination. EMB targets GABA receptors, leading to paralysis and mortality in insects [15]. Although EMB has a shorter half-life than CFP, it is widely distributed in the environment due to its extensive agricultural application. It was reported that EMB residues in surface water, topsoil, and fruits and vegetables were 1.68 µg/L, 30.1 µg/kg, and 0.090 mg/kg, respectively [16,17,18]. EMB has proved toxic in humans and other animals. Symptoms of EMB poisoning include tremors, convulsions, vomiting, and, in severe cases, respiratory failure and paralysis. It was reported that EMB can induce neurotoxicity, hepatotoxicity, developmental toxicity, and nephrotoxicity [19,20,21,22,23]. CFP and EMB often coexist in the environment [24]. However, the effect of CFP and EMB on the kidneys remains unknown. In this study, we aimed to investigate whether individual or combined subchronic exposure to CFP and EMB at relatively low doses causes renal toxicity.

## 2. Materials and Methods

### 2.1. Chemicals

CFP (CAS: 122453-73-0, 98% purity), methoxyamine, and methyl-trimethylsilyl-trifluoroacetamide (MSTFA) were obtained from J&K Scientific Co., Ltd. (Beijing, China). EMB (CAS: 155569-91-8, 97% purity) was purchased from Shanghai Aichun Biotechnology Co., Ltd. (Shanghai, China). Thiobarbituric acid (TBA), trimethylchlorosilane (TMCS), and paraformaldehyde were purchased from Aladdin Biochemical Technology Co., Ltd. (Shanghai, China). Trichloroacetic acid was purchased from Beijing Innochem Technology (Beijing, China). Hydrogen peroxide was purchased from Xilong Scientific Co., Ltd. (Shantou, China). 2,4-Dinitrophenylhydrazine (DNPH) was purchased from Beijing Chemical Corporation (Beijing, China). Guanidine hydrochloride was purchased from Beijing Xinjingke Biotechnology (Beijing, China). Streptomycin sulfate was purchased from Solarbio Science & Technology (Beijing, China). The heptadecanoic acid standard was purchased from CATO Research Chemicals Inc (Guangzhou, China). Hexane and methanol were purchased from Anpel Experimental Technology Inc (Shanghai, China). Trichloromethane was purchased from Beijing TongGuang Fine Chemicals Company (Beijing, China). Pyridine was obtained from Shanghai YiEn Chemical Technology (Shanghai, China).

### 2.2. Animals and Treatment

Male Sprague Dawley rats aged between six and eight weeks and weighing approximately 200 g were acquired from Beijing Vital River Laboratory Animal Technology (Beijing, China). They were kept under specific-pathogen-free conditions with a temperature of 22 ± 2 °C, relative humidity levels ranging from 50% to 60%, and a light/dark cycle of 12 h. The rats had unrestricted access to both water and food. They were randomly assigned to various groups and treated with CFP, EMB, or a combination of these two pesticides. The acute half-lethal oral doses (LD50) for CFP and EMB in rats were 441 mg/kg body weight (BW) and 88 mg/kg BW, respectively [25,26]. In this study, the three low, medium, and high doses were 1, 3, and 9 mg/kg BW/day for CFP and 0.2, 0.6, and 1.8 mg/kg BW/day for EMB, which are 1/450, 1/150, and 1/50 of the LD50 values of each chemical, respectively. The experimental design is shown in Table 1.

CFP and EMB were dissolved in corn oil and given daily to rats via oral gavage for 60 days. Rats in the control group received an equivalent volume of corn oil. All animal procedures were performed according to the applicable Chinese legislation, and the animal protocol was reviewed and approved by the Animal and Medical Ethics Committee of the Institute of Zoology, Chinese Academy of Sciences.

### 2.3. Sample Collection and Preparation

Rats were sacrificed after 60 days of experimental treatment. Blood samples were collected. Serum was obtained by centrifuge at 3000× *g* for 15 min and then stored at −80 °C until it was used for biochemical analysis. Kidney tissues that were snap-frozen in liquid nitrogen and then stored at −80 °C were used for biochemical and metabolomics analysis. The rest of the kidney tissues was fixed with 4% paraformaldehyde for subsequent histopathological identification.

### 2.4. Histopathology

Kidney tissues were embedded in paraffin after being dehydrated by gradient alcohol and xylene. Tissue blocks were sectioned at 5 μm thickness and then rehydrated for hematoxylin and eosin (H&E) and Masson staining. Then, the slides were observed using a microscope.

### 2.5. Serum Biochemical Analysis

Serum creatinine levels were measured by Jeff’s reaction [27]. Briefly, serum proteins were removed by sodium tungstate and sulfuric acid. Creatinine can react with picrate in sodium hydroxide and develop a red-orange color complex. The complex was measured by colorimetric analysis at a wavelength of 520 nm. Blood urea nitrogen (BUN) was measured by a commercial assay kit (Jiancheng Bioengineering Institute, Nanjing, China) according to the manufacturer’s instructions.

### 2.6. Determination of Oxidative Damage Parameters

Oxidation indicators including protein peroxidation and lipid peroxidation and antioxidant indicators including catalase (CAT) and superoxide dismutase (SOD) activities were determined by spectrophotometric methods. Protein carbonyl (PCO), a product of protein oxidation, was measured by reaction with DNPH [28], and malondialdehyde (MDA), one of the small-molecule end-products of the decomposition of lipid peroxidation which was used to reflect lipid peroxidation levels, was determined by reaction with TBA [29,30]. CAT activity was measured by the decomposition rate of H2O2 [31]. SOD activity was measured by the cytochrome c reduction method [32]. A 50% inhibition of the reduction of cytochrome c in a xanthine oxidase coupled reaction system is defined as one unit (U) of the enzyme activity of SOD.

### 2.7. ATP Determination

The levels of ATP were determined by a commercial ATP assay kit (Jiancheng Bioengineering Institute, Nanjing, China) according to the manufacturer’s instructions.

### 2.8. PDH (Pyruvate Dehydrogenase) Activity Determination

PDH activity was determined by a commercial assay kit (Solarbio, Beijing, China), according to the manufacturer’s instructions. In brief, PDH catalyzes the decarboxylation of pyruvate to produce hydroxyethyl thiamine pyrophosphate, which reduces 2, 6-dichlorophenolindophenol (2, 6-DCPIP), resulting in a decreased absorption in 605 nm. The consumption of 1 nmol of 2, 6-DCPIP per minute in the reaction system is defined as one unit (U) of enzyme activity.

### 2.9. Sample Preparation for GC-MS Analysis

The kidney samples were prepared as previously reported [33]. In brief, 100 mg of kidney tissue was homogenized by 600 μL of extracting solution (methanol/chloroform/water = 5:1:1), and then 10 μL of heptadecanoic acid (6 mg/mL) was introduced as an internal standard. Following this, the mixture was centrifuged at 15,000× *g* for 15 min at 4 °C, and the supernatant was collected and then dried in a vacuum concentrator. Fifty microliters (50 μL) of methoxyamine in pyridine was added to the dried metabolite extract and incubated for 16 h at room temperature. Then, 100 μL of derivatization reagent comprising a mixture of MSTFA and TMCS in a 100:1 ratio was added and then incubated for 1 h. Finally, 200 μL of hexane was added and the mixture underwent a final centrifugation at 15,000× *g* for 15 min. The supernatant was collected for GC-MS analysis.

### 2.10. GC-MS Analysis

For the GC-MS analysis, an Agilent 6890N gas chromatograph system (Palo Alto, CA, USA) equipped with an HP-5 MS capillary column (60 m × 0.25 mm × 0.25 μm) was employed. The injection volume used was 2 μL; the injection temperature was 250 °C, and the carrier gas flow rate was 1 mL/min. The temperature of the ion source was 200 °C. The column temperature was held at 90 °C for 1 min and then raised to 175 °C at a rate of 5 °C/min and held for 3 min. Then, the temperature was subsequently raised to 270 °C at a rate of 3 °C/min and raised to 310 °C at a rate of 20 °C/min. The temperature was maintained at 310 °C for 15 min. Ions were generated by a 70 eV in full scan mode, covering a mass-to-charge ratio (*m*/*z*) range of 40–600, with an acquisition rate of 20 spectra/s.

### 2.11. Metabolomics Data Analysis

An Agilent MassHunter workstation was used for deconvolution. Peaks with a signal-to-noise ratio higher than 3 were chosen for further analysis. NIST 2017 was used to identify the metabolites in the samples. Partial least-squares discriminant analysis (PLS-DA) for multivariate statistical evaluation was performed with SIMCA 14.1 software (Umetrics, Umea, Sweden). The differential metabolites were selected by VIP > 1 (calculated by SIMCA 14.1) and *p* < 0.05 (calculated by SPSS 26.0). The heatmap and pathway analysis were generated by the Metaboanalyst website.

To isolate the metabolites that uniquely responded to the conditions tested, the area under the curve (AUC) value for the receiver operating characteristic (ROC) of the differential metabolites was calculated by using SPSS 26.0 software. Metabolites with AUC > 0.9 or AUC < 0.1 were selected.

### 2.12. Statistical Analysis

All data were statistically analyzed by using SPSS 26.0 (Armonk, NY, USA) and are presented as the mean with standard error (mean ± SEM). The difference among groups was determined by one-way ANOVA followed by Duncan’s multiple-range test. *p* < 0.05 was considered statistically significant.

## 3. Results

### 3.1. The Effect of CFP and EMB on Blood Biochemistry

**Figure 1:**
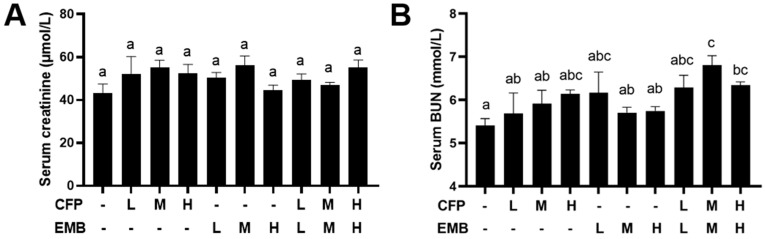
The effect of CFP and EMB on kidney function. SD rats received orally administered CFP at doses of 1, 3, and 9 mg/kg/day and/or EMB at doses of 0.2, 0.6, and 1.8 mg/kg/day for 60 consecutive days. Serum samples were collected after the 60-day exposure and the levels of creatinine (**A**) and blood urea nitrogen (**B**) in serum were measured. Data are presented as mean ± SEM (n = 4). Statistical significance among groups was evaluated using one-way ANOVA, followed by Duncan’s multiple-range test. Different letters indicate significant differences between the groups (*p* < 0.05), while the same letters indicate no significant difference (*p* > 0.05). Abbreviations: CFP, chlorfenapyr; EMB, emamectin benzoate; BUN, blood urea nitrogen; L, low dose; M, medium dose; H, high dose.

To understand whether the two pesticides CFP and EMB induced kidney dysfunction, we determined serum creatinine and BUN. Serum creatinine levels did not exhibit significant variations after the exposure (Figure 1A). BUN levels did not change in the rats exposed to CFP or EMB alone; however, they were significantly elevated in the rats exposed to CFP and EMB in combination at medium and high doses (Figure 1B). The changes in BUN levels suggested that the mixture of CFP and EMB might induce kidney dysfunction.

### 3.2. CFP and EMB Exposure Resulted in Kidney Pathological Changes

**Figure 2:**
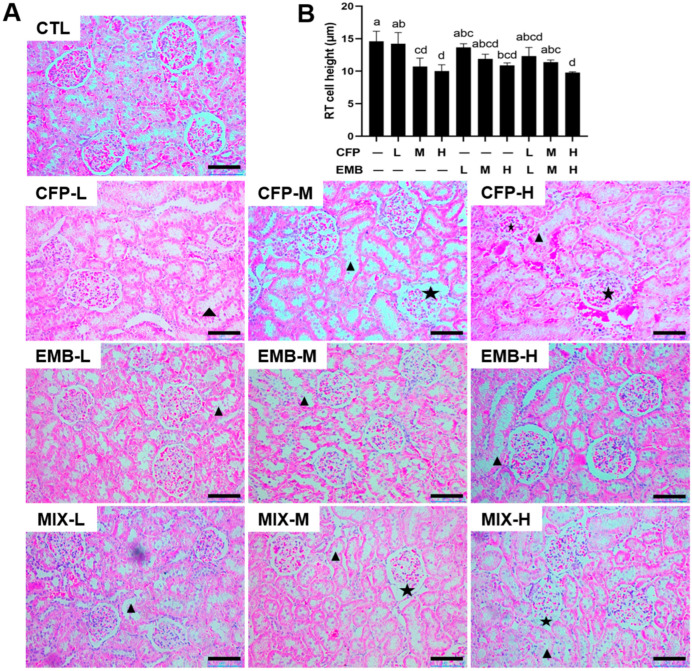
CFP and EMB induce pathological damage in the kidneys of rats. SD rats were treated orally with CFP at doses of 1, 3, and 9 mg/kg/day and/or EMB at doses of 0.2, 0.6, and 1.8 mg/kg/day for 60 consecutive days. Kidney samples were collected after the 60-day treatment. (**A**) Kidney tissue sections were stained by H&E. Scale bar: 100 µm. The triangle indicates thinned renal tubular epithelial cells, and the pentagram indicates an abnormal glomerular morphology. (**B**) Quantitative renal tubule (RT) cell height analysis was calculated by Image J (Version 1.48). Data are presented as mean ± SEM. Statistical significance among groups was assessed using one-way ANOVA followed by Duncan’s multiple-range test. Different letters indicate significant differences between the groups (*p* < 0.05), while the same letters represent no significant difference (*p* > 0.05). Abbreviations: RT, renal tubule; CFP, chlorfenapyr; EMB, emamectin benzoate; MIX, CFP plus EMB; L, low dose; M, medium dose; H, high dose.

**Figure 3:**
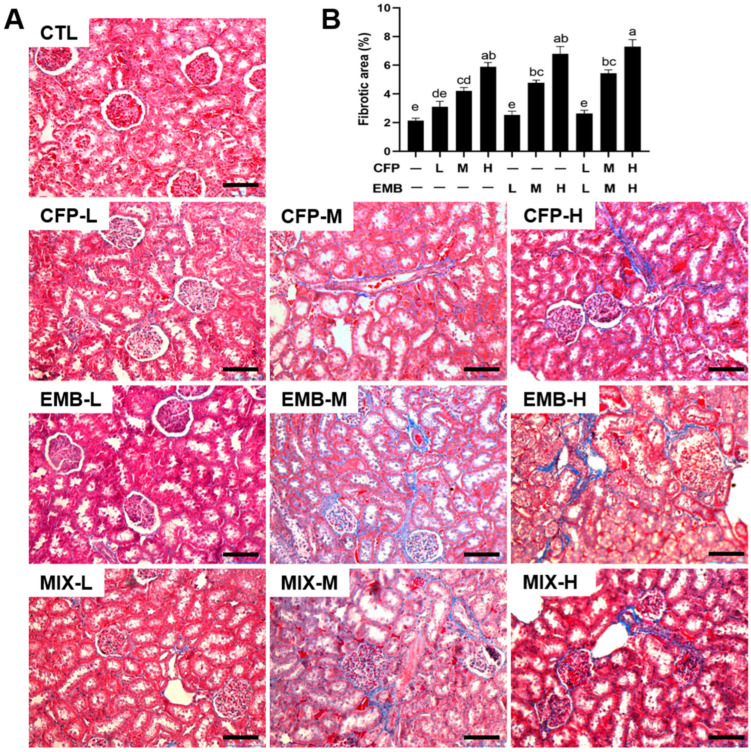
CFP and EMB exposure resulted in fibrosis in the kidneys of rats. SD rats were orally treated with CFP at doses of 1, 3, and 9 mg/kg/day and/or EMB at doses of 0.2, 0.6, and 1.8 mg/kg/day for 60 days. Kidney samples were collected at the end of the 60-day administration. (**A**) Kidney tissue sections were stained by Masson’s trichrome method. Scale bar: 100 µm. (**B**) Quantitative analysis of fibrotic area was calculated by Image J (Version 1.48). Data are expressed as mean ± SEM. Statistical significance among groups was evaluated by one-way ANOVA followed by Duncan’s multiple range test. Different letters indicate a significant difference between the groups (*p* < 0.05), while the same letters indicate no significant difference (*p* > 0.05). Abbreviations: CFP, chlorfenapyr; EMB, emamectin benzoate; MIX, CFP plus EMB; L, low dose; M, medium dose; H, high dose.

We observed the pathological changes in the kidneys. The H&E staining results revealed an irregular glomerular morphology in the samples from the rats exposed to high dose of CFP and medium and high doses of CFP plus EMB (Figure 2A). CFP and/or EMB exposure caused dilated renal tubule lumens with decreased renal tubular epithelial cell height in the kidneys (Figure 2). Masson staining indicated increased fibrotic regions in the kidneys exposed to CFP and EMB alone or in combination, particularly in the medium- and high-dose groups (Figure 3). These findings suggested that individual and combined exposures to CFP and EMB caused significant renal pathological damage.

### 3.3. CFP and EMB Exposure Changed the Kidney Metabolome

**Figure 4:**
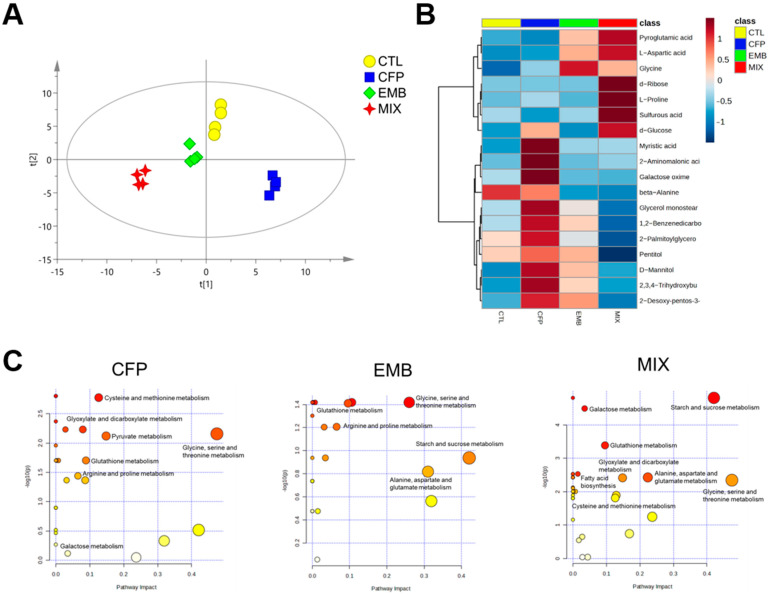
CFP and EMB administration changed kidney metabolic profiles. SD rats were orally treated with CFP at doses of 1, 3, and 9 mg/kg/day and/or EMB at doses of 0.2, 0.6, and 1.8 mg/kg/day. After 60 consecutive days of exposure, kidney samples were collected, and metabolites were analyzed by using the GC-MS method. (**A**) PLS-DA score plots of kidney metabolite profiles of the rats following exposure of CFP (1 mg/kg/day) and/or EMB (0.2 mg/kg/day). (**B**) Hierarchically clustered heatmap of the 18 significantly altered metabolites. (**C**) Significantly affected metabolic pathways. Abbreviations: CTL, control; CFP, chlorfenapyr; EMB, emamectin benzoate; MIX, CFP plus EMB.

To better understand the effects of CFP and/or EMB, we examined changes in the renal metabolome. The results of PLS-DA indicated that the CFP-L, EMB-L, and MIX-L groups were distinct from the control group, suggesting that the renal metabolome was altered after treatment with low-dose CFP and/or EMB (Figure 4A). Subsequently, we identified eighteen differential metabolites with VIP > 1 and *p* < 0.05 (Figure 4B). The majority of these differential metabolites were found to be elevated in rats exposed to low doses of CFP and/or EMB. We conducted ROC analysis on the eighteen metabolites (Table 2). The results indicated that the uniquely significant altered metabolites associated with CFP exposure were 2-aminomalonic acid, 2,3,4-trihydroxybutyric acid, and 2-desoxy-pentos-3-ulose. Glycine was the uniquely significant altered metabolite following EMB administration. The unique altered metabolites observed in rats treated with the combination of CFP and EMB included aspartic acid, sulfurous acid, pentitol, and d-glucose. These findings suggested that the combination of CFP and EMB induced distinct metabolic changes compared with their individual exposure.

Furthermore, we analyzed the differential metabolic pathways following exposure to CFP and/or EMB. Pathway enrichment analysis revealed that CFP and EMB alone or in combination influenced glutathione metabolism (Figure 4C). CFP affected the metabolism of cysteine and methionine, glycine, serine, and threonine, as well as arginine and proline, all of which are related to amino acid metabolism (Figure 4C). Pyruvate metabolism, glyoxylate, and dicarboxylate metabolism, starch and sucrose metabolism, and galactose metabolism, which pertained to carbohydrate metabolism, were disrupted following CFP exposure (Figure 4C). EMB impacted glycine, serine, and threonine metabolism; arginine and proline metabolism; alanine, aspartate, and glutamate metabolism; and phenylalanine, tyrosine, and tryptophan biosynthesis, all associated with amino acid metabolism. Additionally, starch and sucrose metabolism was also influenced by EMB exposure (Figure 4C). The combination of CFP and EMB administration affected starch and sucrose metabolism, galactose metabolism, glyoxylate and dicarboxylate metabolism, and pentose and glucuronate interconversions, all related to glycometabolism (Figure 4C). Moreover, the mixed exposure also impacted glycine, serine, and threonine metabolism; alanine, aspartate, and glutamate metabolism; and cysteine and methionine metabolism, which are linked to amino acid metabolism (Figure 4C). These findings indicated that exposure to CFP and EMB, both alone and in combination, caused significant metabolic alterations in the kidneys, with notable disturbances in amino acid metabolism, especially glutathione metabolism and carbohydrate metabolism. CFP and the mixture of CFP and EMB had a more significant influence on glycometabolism.

### 3.4. CFP and EMB Caused Oxidative Stress in the Kidneys

**Figure 5:**
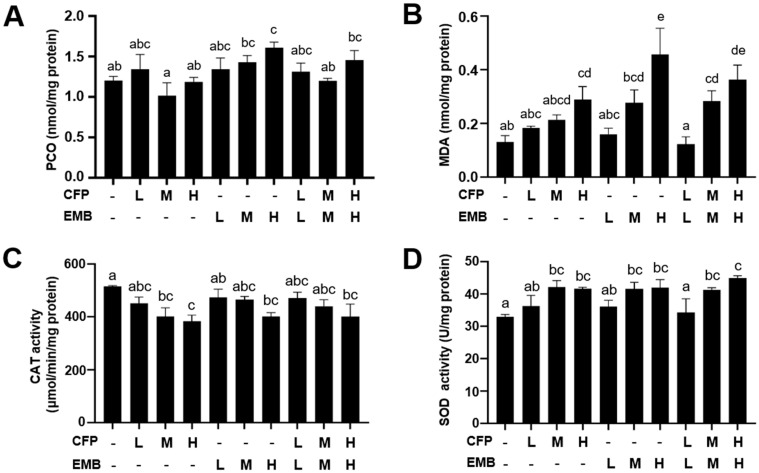
CFP and EMB administration caused renal oxidative stress in rats. SD rats were orally treated for 60 consecutive days with CFP at doses of 1, 3, and 9 mg/kg/day and/or EMB at doses of 0.2, 0.6, and 1.8 mg/kg/day. After exposure, kidney samples were collected, and then the levels of PCO (**A**) and MDA (**B**) and the activities of CAT (**C**) and SOD (**D**) were measured. Data are presented as mean ± SEM (n = 3–4). Statistical significance among groups was evaluated by one-way ANOVA followed by Duncan’s multiple-range test. Different letters indicate a significant difference between the groups (*p* < 0.05), while the same letters indicate no significant difference between the groups (*p* > 0.05). Abbreviations: CFP, chlorfenapyr; EMB, emamectin benzoate; PCO, protein carbonyls; MDA, malondialdehyde; CAT, catalase; SOD, superoxide dismutase; L, low dose; M, medium dose; H, high dose.

To assess the effect of CFP and EMB on oxidative stress in the kidneys, we evaluated the changes in the levels of the peroxidation of proteins and lipids and the activities of the antioxidant enzymes. The results showed that PCO, a well-established indicator of protein peroxidation, was significantly elevated with high-dose EMB administration (Figure 5A). MDA levels, a marker of lipid peroxidation, were markedly increased after exposure to higher doses of CFP and EMB and their combinations (Figure 5B). In addition to oxidative indicators, we also assessed antioxidant enzyme activities in the kidneys. We found that CAT activity decreased while SOD activity increased in the kidneys of the rats exposed to medium and high doses of CFP and EMB individually and in combination (Figure 5C,D). Taken together, these results suggest that exposure to CFP and EMB, alone or in combination, induces oxidative stress, which might be responsible for pathological kidney damage.

### 3.5. CFP Disrupted Energy Metabolism in the Kidneys

**Figure 6:**
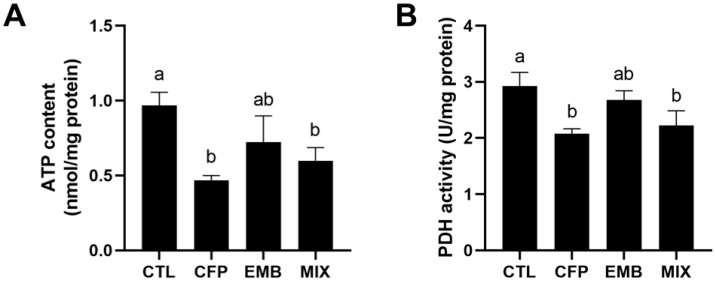
Effect of CFP and/or EMB on energy metabolism. ATP content (**A**) and PDH activity (**B**) were measured in the kidneys of rats exposed to CFP (9 mg/kg/day) and/or EMB (1.8 mg/kg/day) for 60 days. Data are presented as mean ± SEM (n = 4). Statistical significance among the groups was evaluated using one-way ANOVA, followed by Duncan’s multiple-range test. Different letters indicate significant differences between the groups (*p* < 0.05), while identical letters indicate no significant difference (*p* > 0.05). Abbreviations: CFP, chlorfenapyr; EMB, emamectin benzoate; MIX, CFP plus EMB; PDH, pyruvate dehydrogenase.

To understand whether CFP disturbed energy metabolism, we examined ATP content in the kidneys. The results showed that CFP or a mixture of CFP and EMB reduced ATP content, while EMB had no effect on that (Figure 6A). In addition, we measured PDH activity, which was a key enzyme during glucose metabolism and played a crucial role in ATP production. We found that the activity of PDH was inhibited by CFP, which was consistent with the changes in ATP content (Figure 6B). The above results suggested that CFP disrupted energy metabolism in the kidneys.

## 4. Discussion

Residues of CFP and EMB coexist and threaten environmental health [34]. The nephrotoxic effects of EMB have already been reported [19,35], while the nephrotoxicity of CFP was still unknown. Our study showed that combined exposure to CFP and EMB resulted in significant changes in BUN levels, potentially indicating alterations in glomerular filtration function. Correspondingly, notable changes in glomerular morphology were observed following exposure to medium and high doses of CFP and EMB in combination.

Metabolomics technology is extensively applied to investigate the responses to exogenous compounds due to its high sensitivity and correlation with clinical indicators. We explored the renal metabolic response to CFP and/or EMB by using the GC-MS technique and metabolome analysis. PLS-DA score plots indicated that results after exposure to EMB alone were closer to those of the control group compared with exposure to CFP alone or to CFP plus EMB, suggesting that EMB induced a smaller change in the kidney metabolome than CFP and the combination of EMB and CFP. Metabolomics analysis showed that low doses of CFP and EMB caused alterations in the kidney metabolome, although kidney pathological damage was not yet observed. The above results suggest that changes in the metabolome might precede the onset of kidney injury.

ROC analysis revealed that differential metabolites uniquely responsive to CFP were aminomalonic acid, 2,3,4-trihydroxybutyric acid, and 2-desoxy-pentos-3-ulose. Aminomalonic acid, an inhibitor of asparagine synthetase, is derived from cysteine and is recognized as a marker of oxidative stress [36,37]. Consistent with this, CFP influenced amino acid metabolism, especially glutathione metabolism. An abnormal glutathione metabolism usually implies oxidative and antioxidant imbalances. These findings suggested that CFP might induce oxidative stress in the kidneys, as evidenced by increased lipid peroxidation and suppressed CAT activity. Oxidative stress in the kidneys verified the results of metabolome analysis. 2,3,4-trihydroxybutyric acid is involved in ascorbate and aldarate metabolism, a subset of carbohydrate metabolism that is commonly associated with metabolic diseases [38,39,40]. Coincidentally, the results of pathway analysis showed that CFP affected carbohydrate metabolism. CFP inhibited kidney PDH activity, a key enzyme related to glucose metabolism. Carbohydrated metabolism is closely associated with energy supply. The inhibition of PDH activity proved the abnormal carbohydrate metabolism after CFP exposure. PDH was a crucial link between glycolysis and the tricarboxylic acid cycle. When PDH activity is inhibited, less acetyl coenzyme A is produced from pyruvate dehydrogenation. Then, the tricarboxylic acid cycle is hampered in the mitochondria, leading to a reduction in ATP production. ATP produced by carbohydrate metabolism is crucial for maintaining energy metabolism. Our study showed that CFP decreased kidney ATP content, which might be the result of an abnormal carbohydrate metabolism and inhibition of PDH. In addition, another reason for ATP decline in the kidneys might be that CFP could uncouple oxidative phosphorylation. These findings might elucidate the nephrotoxicity associated with CFP.

Glycine was the unique differential metabolite that responded to EMB exposure. Additionally, pyroglutamic also exhibited a response following the administration of low doses of EMB and the combination of CFP and EMB. Glycine and pyroglutamic acid are involved in glutathione metabolism and glycine, serine, and threonine metabolism [41]. These findings were consistent with the changes in differential metabolic pathways after EMB exposure. Disturbances in glutathione metabolism might lead to disruptions in redox balance within the kidneys. The results of PCO, MDA, and CAT proved that EMB induced oxidative stress in the kidneys. Previous studies have demonstrated that subchronic exposure to EMB at a dose of 1/10 of the LD50 induced oxidative stress in the kidneys of mice and rats [2,19]. Conversely, our findings suggested that exposure to EMB at a lower dose (1/50 of the LD50) was sufficient to trigger oxidative stress in the kidneys. Furthermore, EMB affected arginine and proline metabolism. Arginine is a precursor to proline, and metabolites of proline are electrons and reactive oxygen species, leading to oxidative stress [42]. Collectively, the results of metabolomics analysis after EMB exposure were mostly related to oxidative stress, which was confirmed in our study.

Combined exposure to CFP and EMB affected amino acid metabolism, such as glutathione metabolism and glycine, serine, and threonine metabolism, which are related to oxidative stress. Our results validated the finding that combined exposure to CFP and EMB triggered lipid peroxidation and CAT activity inhibition. However, the oxidative damage was not more severe than that observed with exposure to EMB alone. Glucose and pentitol, the unique differential metabolites whose changes we observed in rats exposed to CFP and EMB in combination, are involved in carbohydrate metabolism. Consistent with this, the combined exposure affected galactose metabolism, glyoxylate and dicarboxylate metabolism, starch and sucrose metabolism, and pentose and glucuronate interconversions, which are related to carbohydrate metabolism. The PDH activity and ATP content in the kidneys verified the results of pathways analysis after the administration of the mixture of CFP and EMB. An aberrant carbohydrate metabolism and alterations in PDH activity and ATP content were similar to the effects to exposure to CFP alone, suggesting that these changes after CFP and EMB co-exposure were mainly attributed to CFP. Furthermore, CFP and EMB induced elevated aspartic acid, another differential metabolite that responded to combined exposure to the two pesticides. Aspartic acid has been reported to be a predictor of chronic kidney disease and diabetic nephropathy [43,44]. Therefore, these findings indicated that long-term and low-dose exposure to CFP and EMB may increase the risk of developing chronic kidney disease.

## 5. Conclusions

In this study, we found that CFP and CFP combined with EMB at environmentally relevant levels interfered with renal metabolism, induced oxidative stress, disturbed energy metabolism, and resulted in renal pathological damage. Our findings suggest that long-term exposure to low doses of CFP and EMB may lead to nephrotoxicity.

## Figures and Tables

**Table 1:** Experimental design for combined effects of CFP and EMB.

Groups	CFP (mg/kg/day)	EMB (mg/kg/day)
Control	-	-
CFP-L	1	-
CFP-M	3	-
CFP-H	9	-
EMB-L	-	0.2
EMB-M	-	0.6
EMB-H	-	1.8
MIX-L	1	0.2
MIX-M	3	0.6
MIX-H	9	1.8

Note: SD rats received orally administered chlorfenapyr (CFP) at doses of 1, 3, and 9 mg/kg/day and/or emamectin benzoate (EMB) at doses of 0.2, 0.6, and 1.8 mg/kg/day for 60 consecutive days. CFP and EMB were dissolved in corn oil and administered via oral gavage. The control rats received an equivalent volume of corn oil. Abbreviations: L, low dose; M, medium dose; H, high dose; MIX, CFP plus EMB.

**Table 2:** AUC values of the metabolites that changed significantly in the kidneys of rats.

Metabolites	*p* Value	AUC		
		CFP	EMB	MIX
L-Aspartic acid	0.012	0.625	0.813	1.000
L-Proline	0.000	1.000	0.125	1.000
2-Aminomalonic acid	0.002	1.000	0.406	0.500
beta.-Alanine	0.006	0.375	0.000	0.000
2-Desoxy-pentos-3-ulose	0.025	0.938	0.813	0.375
Sulfurous acid	0.016	0.188	0.500	0.938
2-Benzenedicarboxylic acid	0.002	1.000	0.750	0.063
2,3,4-Trihydroxybutyric acid	0.000	1.000	0.875	0.688
Myristic acid	0.037	0.875	0.500	0.563
Pentitol	0.036	0.500	0.563	0.000
d-Ribose	0.003	0.438	0.375	0.750
d-Glucose	0.008	0.875	0.500	1.000
Galactose oxime	0.017	0.875	0.188	0.219
D-Mannitol	0.035	0.875	0.875	0.438
2-Palmitoylglycerol	0.000	1.000	0.125	0.000
Glycerol monostearate	0.036	0.813	0.438	0.250
Pyroglutamic acid	0.021	0.500	1.000	1.000
Glycine	0.010	0.688	1.000	0.875

Note: *p* values for the comparison of the treatments and control were calculated using ANOVA. AUC values were calculated by using receiver operator characteristic analysis. Metabolites with 0.9 < AUC ≤ 1 or 0 ≤ AUC < 0.1 could effectively discriminate the treatment groups from the control group with high accuracy. Abbreviations: AUC, area under the curve; CFP, chlorfenapyr; EMB, emamectin benzoate; MIX, CFP plus EMB.

## Data Availability

The datasets used and analyzed during the current study are available from the corresponding author on reasonable request. All data generated or analyzed during this study are included in the published article.
